# A comprehensive review on acquisition of phenotypic information of Prunoideae fruits: Image technology

**DOI:** 10.3389/fpls.2022.1084847

**Published:** 2023-01-26

**Authors:** Xuan Liu, Na Li, Yirui Huang, Xiujun Lin, Zhenhui Ren

**Affiliations:** ^1^ College of Mechanical and Electrical Engineering, Hebei Agricultural University, Baoding, China; ^2^ College of Information Engineering, Hebei GEO University, Shijiazhuang, China

**Keywords:** Prunoideae fruits, spectral image, phenotypic information, nondestructive techniques, image technology

## Abstract

Fruit phenotypic information reflects all the physical, physiological, biochemical characteristics and traits of fruit. Accurate access to phenotypic information is very necessary and meaningful for post-harvest storage, sales and deep processing. The methods of obtaining phenotypic information include traditional manual measurement and damage detection, which are inefficient and destructive. In the field of fruit phenotype research, image technology is increasingly mature, which greatly improves the efficiency of fruit phenotype information acquisition. This review paper mainly reviews the research on phenotypic information of Prunoideae fruit based on three imaging techniques (RGB imaging, hyperspectral imaging, multispectral imaging). Firstly, the classification was carried out according to the image type. On this basis, the review and summary of previous studies were completed from the perspectives of fruit maturity detection, fruit quality classification and fruit disease damage identification. Analysis of the advantages and disadvantages of various types of images in the study, and try to give the next research direction for improvement.

## 1 Introduction

Fruit phenotype describes the expression of fruit traits. Research on fruit traits can be done at multiple levels, including cells, tissues, organs, individual fruits, whole plant fruits, and even entire orchards (Dhondt et al., 2013). Phenotypic information of fruit includes but is not limited to geometric size, biomass content, moisture content, and skin color (Shakoor et al., 2017; Choudhury et al., 2019). The variation of phenotypic information is closely related to the market value of fruits. Therefore, it is of great significance to obtain accurate fruit phenotype information for maximizing the economic value of fruits (Mahlein, 2016). Fruit harvesting is an important part of agricultural production. The completion of harvesting at the appropriate fruit harvesting window is the basis for ensuring consumers to obtain high quality fruits. By obtaining phenotypic information such as fruit hardness and SSC (Sohaib Ali Shah et al., 2020), the fruit harvesting window can be accurately grasped, thus guiding the fruit harvesting work. In addition, the research on fruit quality evaluation (Qin et al., 2013; Su and Sun, 2018), fruit disease and damage (Ali et al., 2019) based on phenotypic information is also very meaningful.

In traditional methods, digital calipers and electronic scales were used to measure the size and weight of fruits (Zhang et al., 2014), and Folin-Ciocalteu method (total polyphenol content) and hand-held refractometer (sugar content) (Pissard et al., 2013; Kopjar et al., 2017) were used to determine the polyphenol and sugar content of fruits respectively. These methods are meaningful, but the disadvantages are also obvious, such as the measurement process is time-consuming and damages the integrity of the fruit. With the rapid technological advancement in electronics and computers sectors different technologies were developed to obtain fruit phenotypic information efficiently, accurately and non-destructively. Compared with the traditional detection technology, the phenotypic information detection research based on spectral technology realizes the non-destructive detection of fruit according to the difference between the light absorption rate and the reflectivity inside the fruit (Toivonen et al., 2017). Due to the rich spectral data and image information contained in the images (Fernández-Novales et al., 2019), multispectral and hyperspectral imaging devices have significantly improved the efficiency of fruit phenotype acquisition, and have been widely used by researchers in the related research of fruit phenotype information. The wide applicability of RGB imaging equipment makes the study of phenotypic information based on RGB images a new research hotspot (Blasco et al., 2017). In addition, thermal imaging technology was used in the study of fruit temperature (Osroosh and Peters, 2019; Ranjan et al., 2022), and computer tomography technology (Kritzinger et al., 2017; Karmoker et al., 2018) and laser backscatter imaging technology (Adebayo et al., 2016; Mozaffari et al., 2022) were used in the study of fruit internal quality detection. The phenotypic information acquisition technology based on image technology avoids the measurement error caused by subjective factors in traditional detection methods (Fu et al., 2020), and further improves the accuracy of phenotypic information acquisition. As shown in **Table 1**, the advantages and disadvantages of imaging techniques in the acquisition of fruit phenotypic information and related research were summarized.

**Table 1:** Summary table of imaging technology in the acquisition of fruit phenotypic information and related research.

Research object	Imaging technology	Imaging environment	Portability	Ref.
Cherry	Commercial thermal-RGB sensor	Outdoor environment	Portability	(Ranjan et al., 2022)
Plum	Laser backscattering imager	Closed darkroom t	Non-portability	(Rezaei Kalaj et al., 2016)
Apricot	Laser backscattering imager	Closed darkroom	Non-portability	(Mozaffari et al., 2022)
Peach	Multispectral image system	Closed darkroom	Non-portability	(Herrero-Langreo et al., 2011)
Cherry	UAV Multispectral Imager	Outdoor environment	Portability	(Karydas et al., 2020)
Plum	Hyperspectral imaging system	Laboratory environment	Non-portability	(Li et al., 2018a)
Nectarine	Hyperspectral imaging system	Closed darkroom	Non-portability	(Munera et al., 2018)
Plum	Mobile phones, cameras	Outdoor environment	Portability	(Ahmad et al., 2020)
Cherry	Digital camera	Laboratory environment	Non-portability	(Momeny et al., 2020)

The expression of phenotypic information in fruits may change at different stages of ripening and different kinds of diseases. The researchers found that fungal infection caused an abnormal increase in water content due to the breakdown of carbohydrates (Sun et al., 2018) and a subsequent decrease in chlorophyll content during fruit ripening (Muhua et al., 2007; Lleó et al., 2009). The changes of fruits are closely related to the phenotypic information they present. Therefore, researchers have completed the relevant research on fruits while obtaining phenotypic information based on different types of images.

**Figure 1:**
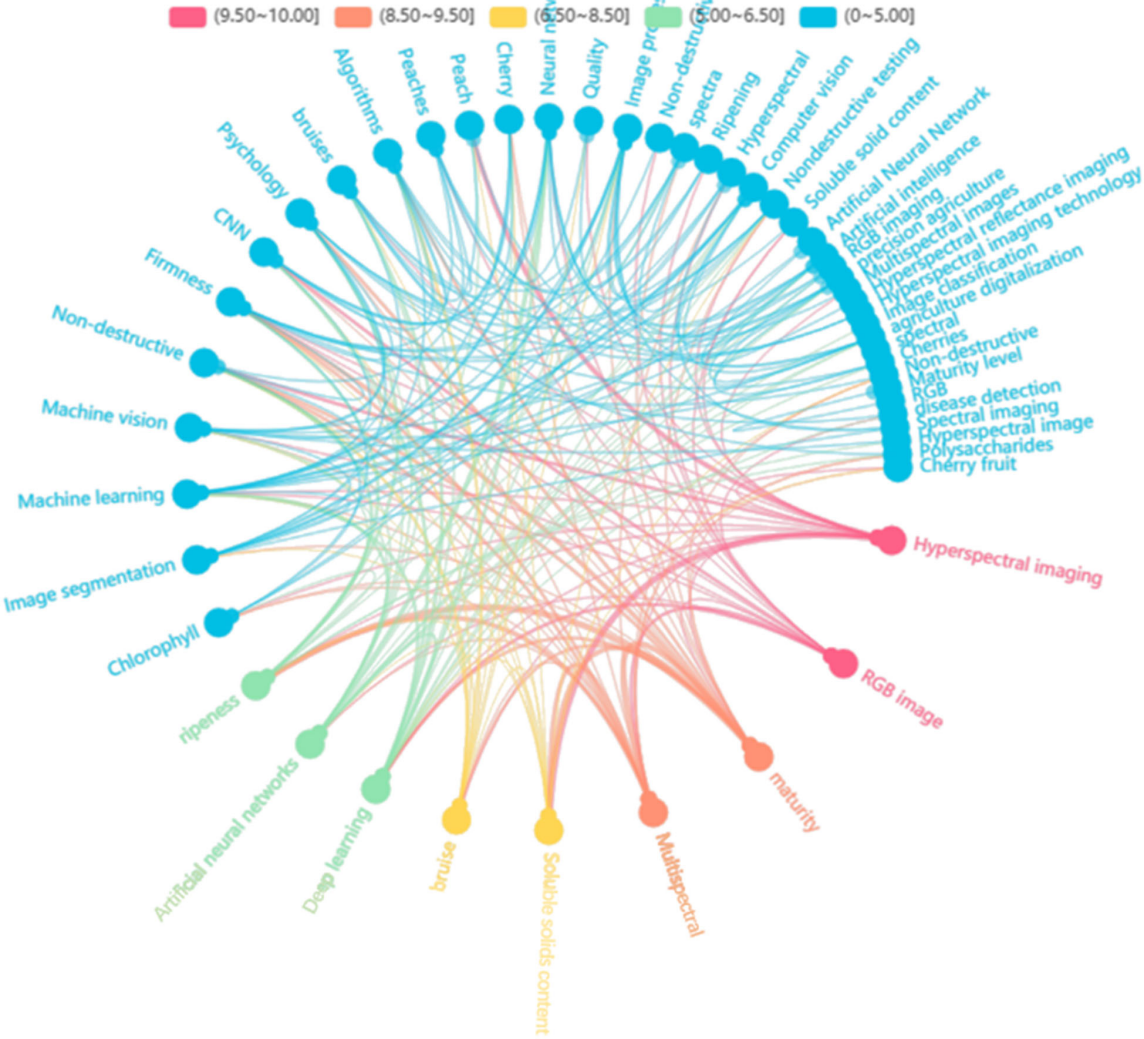
Reference keyword frequency distribution map.

This paper reviews the phenotypic information acquisition and related research based on RGB images, hyperspectral images, and multispectral images, of Prunoideae fruits. The chapter classification is completed according to the image type, and the relevant literature is reviewed and summarized according to the research purposes of fruit quality classification (Pu et al., 2015), disease damage identification (Shao et al., 2019), and maturity detection (Sohaib Ali Shah et al., 2020). The advantages and disadvantages of the completed studies were discussed. Finally, the future trends and challenges of phenotypic information acquisition based on image technology were prospected. Based on the high-frequency words of the cited papers, the word frequency distribution map of the references is drawn. It can be seen from the color degree of the key words in the figure that the related research based on hyperspectral images and RGN images accounts for the majority in quantity. As shown in **Figure 1**, the keyword frequency distribution map of the references in this paper.

## 2 Phenotypic information acquisition and related applications based on RGB image

RGB mode is a color standard, by changing the red (R), green (G), blue (B) three color channels and their superposition to get a variety of colors. RGB mode is one of the most widely used color systems, and RGB images can provide data information such as color features, texture features, and geometric shapes of fruits (Kaur et al., 2018). Compared with multispectral images and hyperspectral images, RGB images can be acquired by smartphones, cameras and other means, and the acquisition methods are more diversified and universal. At the same time, the image acquisition equipment has low requirements on the acquisition environment, even in cloudy days, sunshine or indoor environment (Miragaia et al., 2021).

In recent years, researchers have completed many related studies based on fruit RGB images. This section reviews the research progress of fruit maturity detection, disease damage identification and fruit quality classification based on RGB images.

### 2.1 Fruit maturity detection based on RGB image

As the fruit gradually mature chlorophyll degradation, anthocyanins or carotenoids and other new pigments began to synthesize, resulting in fruit color began to change. Therefore, consumers usually associate color with the ripening stage of fruits (Blasco et al., 2017).

In 2015, S. Taghadomi et al. completed the determination of color parameters during cherry ripening (Taghadomi-Saberi et al., 2015). A CCD camera (PROLINE UK, Model 565s with 510 by 492 pixels resolutions, London, United Kingdom) was used to acquire cherry images. Threshold segmentation technology and Otus algorithm are used to extract cherry image. After screening 37 common features using PCA, 7 features were obtained as input vectors. The relationship between the L^*^ a^*^ b^*^ value measured by the colorimeter and the color features extracted from the cherry image was modeled and analyzed based on the artificial neural network (ANN) using MATLAB. The Levenberg-Marquardt algorithm and trainbr function were used to train the network. The results showed that the ANN with structure of 7-14-11-3 had the best modeling effect on L^*^ a^*^ b^*^ color parameters during cherry ripening (R^2^ = 0.9999). In 2018, Indian scholar Kaur et al. completed a study on the evaluation of fruit maturity based on RGB images of plums (Kaur et al., 2018). The plum images were captured using a digital camera (Nikon Coolpix S3200, Resolution-4608 × 3456, and Format-JPEG) under natural light. In their research, the uniform threshold operator in MATLAB image processing toolbox is used to complete image segmentation.The average RGB value is used to extract color features, and entropy, local binary pattern and discrete cosine transform are used to extract texture features. Based on the multi-attribute decision making (MADM) theory, the decision of maturity level is completed. The results show that the developed system accurately determines the maturity level of plums. The correlation strength between color features and texture features and maturity at different stages is shown in the article (Kaur et al., 2018).

In the following year, researcher Mostafa Khojastehnazhand et al. completed maturity detection and volume estimation based on RGB images of apricot fruit (Khojastehnazhand et al., 2019). They obtained seven kinds of feature information of apricot images, and finally selected G channel, gray value, L^*^ and b^*^ as input features. Discriminant analysis models based on LDA and Quadratic Discriminant Analysis (QDA) are compared. The results show that R^2^ values of LDA and QDA are 0.904 and 0.923 respectively.

There are many ways to obtain RGB images, and the requirements for the environment are not high. However, different acquisition environment and acquisition equipment will make the image have some differences, such as the impact of light conditions, image quality differences. Miragaia et al. completed the research of deep learning algorithm based on convolutional neural network (CNN) to analyze plum maturity detection in real environment (Miragaia et al., 2021). The method of obtaining plum fruit images used in this study is not limited. Plum fruit images obtained by devices such as smartphones and cameras can be used for maturity recognition. The influence of different image quality is solved, so that the obtained images can be used for the detection of fruit maturity. The results show that the developed system has good robustness. Even if the image has different illumination conditions and focus, it can correctly complete the classification of plum fruit maturity, and the efficiency is above 94%. **Table 2** summarizes the studies on fruit maturity detection based on phenotypic information using RGB images.

**Table 2:** Review on fruit maturity detection based on phenotypic information using RGB images.

Research object	Preprocessing	Method	Imaging device	Result	Ref.	
Cherry	Thresholding and Otsu’s algorithm techniques	ANN	CCD camera	R^2 =^ 0.9999	(Taghadomi-Saberi et al., 2015)	
Plum	Uniform thresholding operator	MADM	Digital camera	Correlation between Acidity and Green: R^2 =^ 0.9966; Correlation between R/G and SSC: R^2 =^ 0.8464	(Kaur et al., 2018)	
Apricot	Averaging filter	LDA and QDA	Digital camera	LDA: R^2 =^ 0.904. QDA: R^2 =^ 0.923	(Khojastehnazhand et al., 2019)	
Plum	Mean image subtraction, mean pixel subtraction	Deep Learning	Mobile phone, camera	Maturity recognition accuracy>94%	(Miragaia et al., 2021)	

It can be concluded from **Table 2** that the related research on maturity detection and classification completed by researchers using RGB images has achieved good research results. The reason for the analysis is that the plum fruits studied are all varieties with strong correlation between maturity and fruit color changes, and RGB images can better reflect the color characteristics of fruits at different maturity stages.

### 2.2 Fruit quality detection based on RGB image

**Figure 2:**
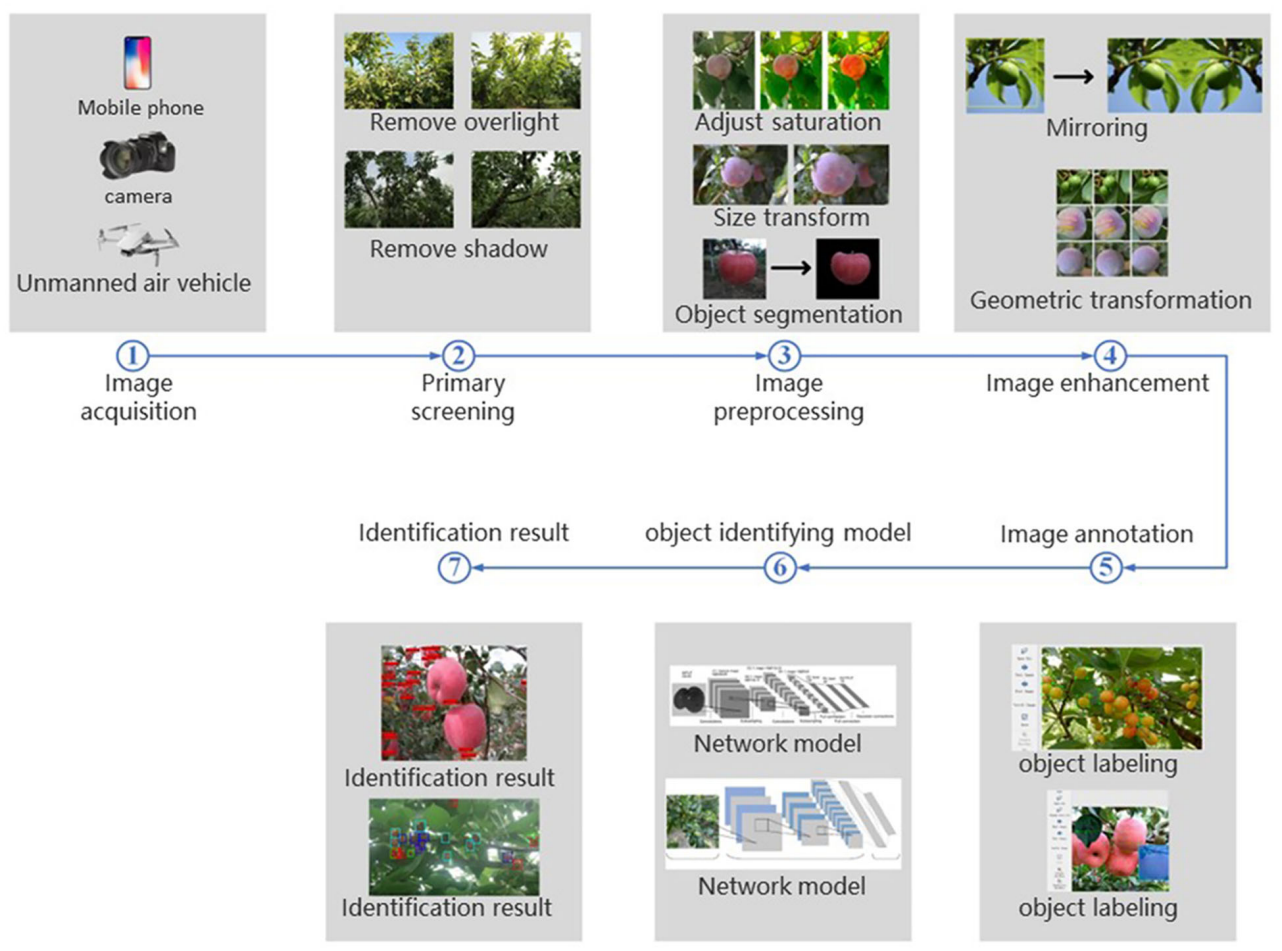
Image processing steps in target recognition.

In the field of fruit quality detection, the quality detection based on RGB images is more focused on the phenotypic information obtained from the fruit surface. This is because the fruit RGB images only contain surface phenotypic information such as color features, geometric features, and texture features. We try to give the steps followed by common image processing in target detection based on RGB images (**Figure 2**). It should be pointed out that the processing steps mentioned are not necessarily used, and the specific methods are related to the actual needs.

Iranian researcher Esehaghbeygi et al. used a high-resolution CCD (PROLINE UK, Model 565s with 510 by 492 pixels resolution) camera to obtain RGB images of peach fruit, and completed the study of color grading and size evaluation of peach fruit (Esehaghbeygi, 2010). An edge detection algorithm was developed to estimate the volume of peach fruit, and the Mesh function in MATLAB software was used to obtain the hue, saturation and value of peach image and complete the classification of peach fruit color and the detection of surface spots. The results show that the detection rate of white spots is 96.7%, the detection rate of brown spots is 85%, the accuracy of size classification is 96%, and the accuracy of color classification is 90%. It is an unavoidable problem to eliminate the influence of other light in the detection of fruit quality in outdoor environment. Wang et al. completed a study on the color evaluation of sweet cherry (Wang et al., 2012). The acquisition of cherry images was completed by a digital camera (Nikon D5000 Nikon Inc., Melville, NY, USA). The acquisition environment was an outdoor environment with sufficient light. The cherry images of direct sunlight, bright shadow and dark shadow were obtained respectively. The distance between the camera and the cherry was 0.5 m. Using image processing technology to eliminate the strong light spots caused by light, mainly includes two steps: (1) using the green channel of color grading area to detect the image pixels with dazzling reflection; (2) Eliminate detected pixels from the color rating area of the image. In the study, a color rating system was successfully developed to complete the color rating of cherry images. The results showed that the overall accuracy of color rating was more than 85%.

Taghadomi-Saberi ‘s team combined image processing technology and ANN to complete the evaluation of antioxidant activity and anthocyanin content of sweet cherries (Taghadomi-Saberi et al., 2014). A color CCD camera (PROLINE UK, Model 565S, London, United Kingdom) was used to obtain the cherry image. The Otsu method is used to complete the cherry image segmentation. Two prediction models based on ANN and adaptive neuro-fuzzy inference system (ANFIS) were established and compared, and the accuracy of the two models for antioxidant activity and anthocyanin content was evaluated. The results showed that the prediction models with the structure of 11-14-9-1 and 11-6-20-1 based on ANN had the highest correlation coefficients with antioxidant activity and anthocyanin content, which were R = 0.93 and R = 0.98, respectively. Meanwhile, the ANFIS prediction model obtained the best prediction results (R = 0.87 and R = 0.90) when using triangular and two-term Gaussian membership functions. Cherry fruit skin color as an important indicator to measure its maturity and quality has become a research hotspot. Researchers (Wang et al., 2012; Taghadomi-Saberi et al., 2014; Taghadomi-Saberi et al., 2015) have studied color as an important indicator in their studies, but have neglected the effect of cherry shape appearance on its quality. In 2020, Mohammad Momeny ‘s team completed the analysis of cherry quality from the perspective of cherry shape appearance (Momeny et al., 2020). They used an improved CNN to detect the appearance shape of cherries. The classification algorithm completed the classification of regular and irregular cherries with an accuracy of 99.4%. By using mirroring, rotation and other methods to expand the data set, 14,380 images are finally obtained. It is necessary to establish a perfect data set to obtain higher classification accuracy. In the study, the deep CNN based on hybrid pooling was compared with KNN, ANN, Fuzzy, and integrated decision tree methods based on histogram of gradient and local binary pattern feature extraction methods. The results show that the improved CNN method is suitable for the detection of cherry appearance (regular and irregular shaped).

In the review of reported studies, we found that building a complete and sufficient image data set is a prerequisite for obtaining accurate prediction results. Villacrés, a researcher in Chile, installed an RGB camera on a tractor through a self-stabilized pan-tilt for image acquisition. The camera is 1m away from the cherry tree, and a total of 15,000 images are obtained for the construction of the data set, which greatly improves the efficiency of image acquisition (Villacrés and Cheein, 2020). Four different color borders were used to label the cherries in advance and different colors were used to distinguish the size of the cherries. Faster R-CNN meta-architecture and Inception V2 were used as feature extractors for the detection of cherries. The results show that the cherries in the image can be identified with an accuracy of 85% and the cherries are divided into four sizes according to the requirements of farmers. The main reason why cherries are not detected in the study is that the fruit pixels caused by occlusion are too small to detect (pixels less than 20). **Table 3** summarizes the studies on fruit quality detection based on phenotypic information using RGB images.

**Table 3:** Research summary of fruit quality detection based on phenotypic information using RGB images.

Fruit types	Imaging environment	Research aim	Research method	Result	Ref.
Peach	Standard Lighting Room	Color rating; Volume estimates	Edge detection algorithm	Detection rate of white and brown spots: 96.7%, 85%; Size and Color Classification Accuracy: 96%, 90%	(Esehaghbeygi, 2010)
Cherry	Outdoor with sufficient light	Color rating;	Color Rating System	Color Rating Accuracy: >85%	(Wang et al., 2012)
Cherry	Uniform illumination environment	Antioxidant activity; Anthocyanin content	ANN, ANFIS	Triangular: R=0.87; Gaussian membership functions: R=0.90	(Taghadomi-Saberi et al., 2014)
Cherry	Inside the lighting box	Appearance shape classification	CNN	Classification accuracy: 99.4%	(Momeny, et al., 2020)
Cherry	Outdoor in different light conditions	Identification and size grading	Faster R-CNN	Recognition rate: 85%	(Villacrés and Cheein, 2020)

Through the analysis of **Table 3**, we can conclude that the current research on fruit color quality grading and fruit size estimation based on the color features and geometric size features of RGB images has achieved high accuracy. In the above research, the recognition and classification accuracy based on CNN is more satisfactory. Researchers have further improved the research accuracy based on CNN by innovating on the infrastructure. However, in the research of small fruit recognition with small fruit volume and complex image acquisition environment, improving the recognition detection rate is still the focus of the next research.

### 2.3 Fruit disease damage detection based on RGB image

CNN method widely used in research related to visual recognition, such as image classification (Ahmad et al., 2017), target detection and recognition (Ahmad et al., 2018b), and image matching (Ahmad et al., 2018a). In the previous chapters, some researchers have also reported the use of CNN to complete related research on fruit quality. This chapter summarizes the related research on fruit disease image classification and recognition based on RGB image, including fruit disease image acquisition method, image preprocessing technology and neural network algorithm. The feature information contained in RGB images is mostly the phenotypic information existing in the fruit surface, such as fruit color, size and other feature data. Therefore, the fruit disease detection based on RGB images is mainly aimed at the disease and damage of the fruit surface layer.

In a study on plum disease detection reported in 2020, researcher Ahmad et al. proposed a plum disease detection framework based on CNN (Ahmad et al., 2020). Four structures are compared in the study: AlexNet, VGG-16, Inception and Resnet. The data set is expanded and made more challenging through data augmentation to achieve robust model training. The recognition accuracy of the model before and after data enhancement is compared. The experimental results show that the performance of the model increases with the increase of the number and complexity of the data set. The research on different architectures shows that the disease identification and classification results based on Inception-V3 model are the best, reaching 92%. In order to run on resource-constrained devices, Jamil Ahmad et al. quantized the Inception-v3 model from FP32 precision to FP16, gaining a 2× speedup and 2× less memory requirement.

Huang et al. completed a peach fruit RGB image disease detection method based on asymptotic non-local means (ANLM) image algorithm and parallel convolutional neural network fusion in 2020 (Huang et al., 2020). Firstly, ANLM is used to remove the interference of complex background in the image, and then parallel convolutional neural network is used to identify peach disease features. In the research, the improved elu activation function is used to replace the traditional Eelu activation function, and the linear particle swarm optimization ELM proposed in the research is used to replace the traditional softmax layer. Through the improvement of the algorithm, the convergence speed and accuracy of the network are significantly improved. The results showed that the accurate detection rates of brown rot, black spot, anthracnose, scab and normal peach were all above 85%, indicating that the improved parallel convolutional neural network algorithm was an effective method for peach disease detection.

The segmentation algorithm greatly affects the segmentation accuracy, and accurately obtaining the region of interest is the premise of accurately identifying the type of disease defects. Therefore, Alosaimi et al. proposed a new CNN model for detecting peach disease categories (Alosaimi et al., 2021). In the study, Mask R-CNN was used to complete the segmentation of the diseased area, and then the VGG-19 architecture was used to identify the type of the segmented area. The peach disease database in the study consists of a public database and photos obtained in the natural environment. The photos obtained in the natural environment include different types such as direct sunlight and cloudy days. Finally, the mean Average Precision (mAP) was used to evaluate the performance of the model. The results showed that the improved CNN for peach disease classification had mAP = 94%.

Deep learning is widely used in related research using imaging data to detect disease categories, but it is difficult to collect a large number of peach disease images, and the sample images are unbalanced. In response to this problem, Yao et al. proposed an improved Xception network called L2MXception (Yao et al., 2021). The network integrates regularization terms of L2 norm and mean. In the study, the recognition results of seven deep learning models were compared, and the composition of the peach disease image dataset included seven disease types. The results show that the classification accuracy of L2MXception reaches 93.85%, which is 28.48% higher than that of Xception model.

Based on the above review, we found that various machine learning algorithms have been applied to the research field of fruit tree disease recognition. Compared with the traditional technology, the application of new technology greatly improves the detection speed and recognition classification accuracy. Obtaining rich and complete data sets is necessary for the establishment of models with excellent performance. This puts forward higher requirements for the acquisition efficiency of image data sets, and automatic, efficient and reliable data acquisition equipment becomes very important.

### 2.4 Other related research based on fruit RGB image

Based on the feature information obtained from RGB images, researchers have completed related research on fruit maturity, quality detection, disease damage and other fields. In addition, it was also reported that relevant researchers completed other related studies based on fruit RGB images.

In 2021, Ropelewska completed the study of cherry variety discrimination using the acquired cherry images (Ropelewska et al., 2021). The research proves that the discriminant model based on texture parameters obtained from different color channels and texture parameters obtained from different color spaces has high recognition accuracy for cherry varieties. Discriminant models based on histogram, co-occurrence matrix, run-length matrix, autoregressive model and gradient map are considered in the experiment. The results show that the accuracy of the model based on the texture parameters selected from the color space is slightly higher than that of the texture parameters obtained by inputting the color channel. In the color channel R, X and color space, the accuracy of the texture parameters based on histogram and co-occurrence matrix to distinguish the three sweet cherries reached 100%. In the color channel, the histogram model based on the color channel L produces the highest accuracy of 97%. Similar studies have also been applied to the field of peach variety identification. Ropelewska et al. compared the classification results of different color channels and different discriminant models by acquiring images of peach skin, flesh, stone and seed texture. (Ropelewska and Rutkowski, 2021). The results show that the texture features based on different color channels can better complete the identification of peach varieties.

## 3 Phenotypic information acquisition and related applications based on hyperspectral image

Hyperspectral imaging technology has broad application space in the field of agriculture, and has gradually become one of the important and cutting-edge technical means in agricultural applications. The application of hyperspectral images includes crop growth monitoring, crop stress monitoring (Chattaraj et al., 2013), crop yield primary estimation (Ye et al., 2006), vegetation coverage monitoring, and non-destructive monitoring (Huang et al., 2014) of agricultural products, providing service support for precision agriculture and agricultural management. Compared with multispectral images, hyperspectral imaging equipment has a wider range of imaging wavelengths, which greatly improves the information richness of hyperspectral images. In the processing technology and application, the acquisition of rich spectral data makes more reasonable and more effective analysis possible. Therefore, hyperspectral image technology has incomparable development potential. Hyperspectral imaging equipment is mainly composed of halogen light source, imaging lens, computer, transmission platform, transmission motor and other parts.

### 3.1 Fruit maturity and biochemical parameters detection based on hyperspectral image

Hyperspectral imaging equipment obtains spectral data of hundreds or thousands of samples with nanoscale sampling resolution. There is inevitably a large amount of redundant data in the rich spectral data, and more redundant data usually lead to longer and unreliable prediction of dependent variables. Therefore, eliminating too much redundant data has become an inevitable process. Researchers have used many variable selection methods, such as ANN, partial least squares (PLS), principal component analysis (PCA), genetic algorithms (GA), etc. For details on variable selection methods, please refer to Xiaobo et al. (Xiaobo et al., 2010). There is no accurate range for the research of fruit quality, and the existing related research focuses on fruit SSC, acidity, firmness, etc. These parameters are directly related to the fruit flavor tasted by consumers.

Munera et al. obtained hyperspectral images of peach fruit ripening process with a wavelength range of 450-1040 nm using laboratory hyperspectral imaging equipment (Munera et al., 2017). Internal Quality Index (IQI) and Ripening Index (RPI) were introduced to evaluate peach fruit maturity. Variable Importance in Projection (VIP) was used to complete the spectral screening of hyperspectral images, and the regression models of IQI and RPI were established based on PLS regression analysis. Based on the spectral data of each pixel, the IQI and RPI prediction values of the peach fruit image were obtained, and the visualization of the peach fruit maturity distribution map was realized. The results showed that in the prediction of IQI and RPI of the two varieties of peach fruit, the R^2^ values of the two indexes and the two varieties were greater than 0.87. Li et al. completed a study on the detection and classification of SSC and PH content and maturity of cherry fruits based on near-infrared hyperspectral imaging technology (Li et al., 2018e). The wavelength range of hyperspectral image of cherry fruit is 874-1734 nm, and the distance between lens and sample is 30.5 cm. In order to accurately complete the classification of cherry maturity, the samples were classified by five orchard owners according to the principle of majority, and the classification model of cherry maturity was established by supervised learning method Linear Discriminant Analysis (LDA). According to the spectral characteristic parameters of different maturity categories, the classification of cherry maturity was completed. The results show that the accuracy of the classification model is 96.4%.

The relevant literature shows that the research methods based on hyperspectral images have been widely used in the research of fruit maturity, including the optical index (Lleó et al., 2011) based on spectral image acquisition for maturity detection and grading, and the maturity prediction and grading based on the established prediction model (Munera et al., 2017; Li et al., 2018e). The summary of related research in recent years is completed through **Table 4**.

**Table 4:** Summary of research on maturity detection based on hyperspectral imaging.

Research Objective	Research object	Method	Wavelength coverage	Wavelength screening	Optical index	Model	Ref.
Fruit Maturity Detection of Prunoideae	Peach	Optical index	640, 675, 680, 720, 730, 800 nm	/	Ind1, Ind2, Ind3, IAD	/	(Lleó et al., 2011)
	Peach	Forecasting model	380-1030 nm	SPA, UVE, CARS	/	PLSR, LS-SVM	(Zhu et al., 2016)
	Peach		450-1040 nm	VIP	/	PLS	(Munera et al., 2017)
	Cherry		874-1734 nm	GA, SPA	/	LDA	(Li et al., 2018e)

From the **Table 4**, we found that the wavelength range used in the study was mostly concentrated near the chlorophyll absorption peak and SSC absorption peak. This is because the content of biochemical substances in the fruit will change with the change of maturity. The feasibility of fruit maturity classification by detecting the content of biochemical substances has been verified by relevant researchers. However, there are some differences in wavelength selection among different varieties.

In 2017, Zhu et al. obtained hyperspectral images of different slices inside peaches using visible and short-wave near-infrared spectral imaging equipment (380-1030 nm) and long-wave near-infrared spectral imaging equipment (874-1734 nm) (Zhu et al., 2017). Savitzky-Golay smoothing and standard normal variate transformation were used to preprocess the spectral images. Subsequently, PLSR and LS-SVM modeling methods were established and compared. In their study, the contents of protopectin, water-soluble pectin and total pectin were predicted respectively, and results showed that the prediction model had better prediction results for protopectin than for water-soluble pectin. According to this result, it was analyzed that water-soluble pectin dissolved in the process of destructively obtaining peach slices. The visualization of peach fruit pectin content distribution was also completed in the study. Pectin content is considered to be closely related to maturity. Pectin distribution map can be used as an indicator to provide guidance for understanding fruit ripening and post-harvest storage systems. In the subsequent study, Li et al. completed the detection of non-destructive quality attributes of plum fruit from three quality indicators: color, firmness and SSC (Li et al., 2018a). Two hyperspectral cameras with wavelength ranges of 600-975 nm in visible and near infrared (VNIR) region and 865-1610 nm in short wave near infrared (SWIR) region were used to obtain hyperspectral images of two varieties of plum fruits. Because the surface of plum fruit is smooth and has strong reflection, which is different from peach fruit, the intensity of fruit edge in the sample spectral image is low. They cited the automatic correction method for light scattering of spherical objects developed by Gomez-Sanchis et al. to improve this phenomenon (Gómez-Sanchis et al., 2008). At the same time, a PLSR model was established for the non-destructive measurement of firmness, SSC and color components of two different plum varieties. The results showed that there was a strong correlation between SSC and SWIR spectra, and the predicted correlation coefficient r_p_^2^ was greater than 0.8. The VNIR spectrum has a good correlation with color, and the r_p_^2^ value is greater than 0.7 for L^*^ and a^*^. Both hyperspectral imaging systems have low prediction accuracy for hardness.Shen et al. predicted the SSC of green plum using sparse autoencoder (SAE) in a study reported in 2020 (Shen et al., 2020). SAE is an unsupervised machine learning algorithm, which continuously adjusts the autoencoder parameters and finally completes the model training by calculating the error between the autoencoder output and the original input. The hyperspectral imaging equipment used in the experiment has a spectral range of 400-1000 nm and a spectral resolution of 2.8 nm. A multi-layer network model SAE-PLSR was proposed to predict SSC, and the sparsity parameter ρ was set to 0.01 (Tang et al., 2016). At the same time, BP, SVR, PLSR, SAE-BP, SAE-SVP and SAE-PLSR were compared. The results showed that SAE-PLSR model had the best prediction results, r and root mean square error (RMSE) of prediction set were 0.938 and 0.654 respectively.

With the improvement of deep learning theory, Yang et al. introduced deep learning theory into the prediction of SSC content of peach, and proposed a SSC estimation method of fresh peach based on deep features of hyperspectral image fusion information (Yang et al., 2020). The distance from the peach sample to the lens is 220 mm, the wavelength range is 900-1740 nm, and the spectral resolution is 5 nm. In the research, the stacked autoencoder is used for the depth feature of the spectrum and the depth feature extraction of the image. Unlike the sparse autoencoder used by Shen et al. (Shen et al., 2020), the stacked autoencoder is a cascade of multiple autoencoders to complete the task of layer-by-layer feature extraction. The resulting features are more representative and have a small dimension. Finally, a stack autoencoder - random forest peach SSC estimation model based on hyperspectral image fusion information depth features was established. The results show that the estimation model with the network structure of 1237-650-310-130 has the highest accuracy, the training set R^2^ = 0.9184, and the validation set R^2^ = 0.8838. In 2022, Xuan et al. completed the analysis of SSC, firmness, diameter, weight and other internal and external quality of peach fruit based on hyperspectral images (Xuan et al., 2022). The wavelength range of hyperspectral image is 400-1000 nm, and the distance between the sample and the lens is set to 47 cm. The CARS and random frog algorithms were used to select effective wavelengths, and a non-destructive regression model for predicting SSC and firmness was established based on multiple linear regression (MLR). The results showed that the CARS-MLR model had a good prediction effect on SSC, and the training set and validation set were R^2^_C_ 0.856 and R^2^_V_ 0.841, respectively. In the study, the estimation information of fruit size and weight was obtained by extracting pixel diameter and area, the estimation of peach diameter was completed by using the minimum boundary rectangle method, and the weight of peach fruit was predicted by MLR model. The results showed that the maximum error was 3.14 mm, the average absolute error was 0.94 mm and the average percentage error was 1.01%. The MLR regression model was established for weight estimation. The training set and validation set were R^2^_C_ = 0.946 and R^2^_V_ = 0.957, respectively.

Li et al. completed a study on the detection and classification of SSC and PH content and maturity of cherry fruit based on near-infrared hyperspectral imaging technology (Li et al., 2018e). The research content related to maturity detection and classification has been summarized in the previous chapters. Here, the related research on SSC and PH content is reviewed. The cherry samples studied completed the establishment of calibration set and validation set according to the principle of interval sampling. Principal components regression model and PLSR model based on full spectrum and GA-MLR model based on characteristic bands were established. For the prediction model established by full spectral band, the prediction result of SSC is better than that of PH, the reason is that the fluctuation range of PH value is too small, and the PH value only fluctuates between 3.3 and 4.1. The GA and SPA wavelength selection algorithm are compared in establishing the prediction model with characteristic band as input. The results show that the prediction model of feature wavelength screening by GA has achieved better results. The prediction evaluation indexes of GA-MLR model are shown in the article. **Table 5** summarizes the application of fruit quality detection based on phenotypic information through hyperspectral images in recent years.

**Table 5:** Summary of fruit quality detection based on phenotypic information from hyperspectral imaging.

Research Object	Biochemical parameter	Imaging environment	Wavelength coverage	Wavelength screening	Model	Evaluating indicator	Ref.
Peach slices	Protopectin, Water-soluble pectin and Total pectin	Laboratory environment	380-1030 nm and 874-1734 nm	SPA, UVE, CARS	PLSR, LS-SVM	Protopectin: RPD=2.264; Water-soluble pectin and Total pectin are poor predictors	(Zhu et al., 2017)
Peach	SSC	Sealed black box	325-1100 nm	MC-UVE, CARS, RF	PLS	Set-I: r_p_=0.9192, RMSEP=0.3967 Set-III: r_p_=0.8469, RMSEP=0.4260	(Li et al., 2018d)
Plum	Color, firmness, SSC	Laboratory environment	600-975 nm and 865-1610 nm	PCA	PLSR	SSC: r_p_^2^>0.8 L^*^ and a^*^: r_p_^2^>0.7	(Li et al., 2018a)
Green plun	SSC	Laboratory environment	400-1000 nm	SAE	BP, SVR, PLSR, SAE-BP, SAE-SVP, SAE-PLSR	SAE-PLSR: r_p_=0.938; RMSEP=0.654	(Shen et al., 2020)
Peach	SSC	Sealed black box	900-1740 nm	Stacked Autoencoder	random forest	Calibration set R^2 =^ 0.9184; Validation set R^2 =^ 0.8838	(Yang et al., 2020)
Cherry	TSS and firmness	Dark room	500-1500 nm	VIP	PLSR, GPR	GPR: TSS: RPD_T_=3.4, R^2^_T_=0.88, RMSE_T_=0.43%; Firmness: RPD_T_=2.54, R^2^_T_=0.60, RMSE_T_=0.38N; PICP: 0.90-0.97	(Pullanagari and Li, 2020)
Peach	SSC, firmness, diameter, weight	Laboratory environment	400-1000 nm	CARS and RF	CARS-MLR; MLR	SSC: R^2^_V_: 0.841, RMSEV: 0.546; RPD: 2.51, Firmness: R^2^_V_: 0.826; RMSE_V_: 1.008, RPD: 2.401 Weight: R^2^_v_=0.957, RMSE_V_: 9.203, RPD: 4.819	(Xuan et al., 2022)
Cherry	SSC and PH	Sealed black box	874-1734 nm	SPR; GA	PCA; PLSR; GA-MLR; SPA-MLR	GA-MLR: SSC: R_C_^2 =^ 0.897, RMSEC= 1.054, R_P_^2 =^ 0.863, RMSEP=1.210 RPD=2.700	(Li et al., 2018e)

Through **Table 5**, it can be seen that the acquisition environment of fruit hyperspectral images required by researchers still stays in a relatively stable acquisition environment such as laboratory and closed dark box. Such an environment does not require strong mobility of imaging equipment, and significantly reduces the impact of external light sources on images.

### 3.2 Fruit disease damage detection based on hyperspectral image

Many researchers have focused on the use of hyperspectral imaging technology to detect fruit phenotype information to achieve damage detection for fruit diseases, including disease identification, classification and quantification (Du et al., 2020). Compared with traditional disease damage detection, hyperspectral imaging technology provides an efficient non-destructive detection method (Wang et al., 2015). This section mainly introduces the application of hyperspectral image in prunoideae fruit disease damage detection.

The types of fruit disease damage are diverse and complex. Due to the existence of disease damage, the phenotypic information expressed by the fruit is abnormal, which reflects the abnormal spectral characteristics on the spectral image. In 2016, Li et al. completed the detection and recognition of nine defects in a two-color peach based on hyperspectral images (Li et al., 2016). The wavelength range of the hyperspectral image is 325-1100 nm, the spectral resolution is 2.8 nm, the distance between the sample and the lens is set to 400 mm, and the image acquisition environment is a closed darkroom. Principal component regression and PCA was used to complete the wavelength selection of hyperspectral image. In the study, according to the weight coefficient of each band, the spectral wavelength image corresponding to the maximum difference of weight coefficient is selected to obtain the dual wavelength ratio image and complete the detection of disease damage. The results show that the defect recognition effect of ratio image is better than that of single wavelength image, and the total detection rate of nine defects is 96.6%. In the subsequent study, the Li ‘s team completed the detection of early bruises on peaches (Li et al., 2018c). Accurate non-destructive testing of early bruises is a challenging task. Due to being in the early stage of bruises, peaches have not significantly improved in appearance. In the study, they used two hyperspectral spectrometers for image acquisition, with spectral ranges of 325-1100 nm and 930-2548 nm, respectively, and used PCA to complete the wavelength screening of hyperspectral images. They proposed an improved watershed segmentation algorithm and compared it with the traditional Ostu segmentation algorithm and global threshold segmentation method. The results of the study showed that the accurate recognition rate of damaged peaches reached 96.5%, and the accurate recognition rate of healthy peaches reached 97.5%. Their proposed method of using hyperspectral images combined with improved watershed segmentation algorithm to detect early peach bruises is an effective method that can accurately and non-destructively detect early peach bruises.

Peach is a perishable fruit, in order to extend the storage time of peaches, peaches are usually stored at low temperature. Peaches will suffer from freezing damage due to low temperature for a long time, resulting in a decrease in their market value. The symptoms are peach fruit texture deterioration and lack of juice. Pan et al. established a hyperspectral imaging system based on laboratory environment to detect cold injury of peach (Pan et al., 2016). The wavelength range of hyperspectral image used was 400-1000 nm, and the lens was 400 mm above the sample. The discrimination model of peach fruit cold injury was established by using multi-layer perception artificial neural network (MLPANN). The comparison test of full spectrum input and eight optimal wavelengths input was completed. The results showed that the overall classification accuracy of MLPANN peach cold injury discriminant model based on characteristic wavelength was 92.9%. Pan et al. also proposed the cold injury (CI) index to quantify the frostbite degree of peach fruit. The formula for obtaining the cold injury (CI) index is as follows: CI index = [(CI score) × (number of fruits with this CI score)]/(3 × total number of fruits). Sun, a member of the same team, studied chlorophyll content to detect decayed honey peaches in the following year (Sun et al., 2017). In this study, the wavelength range of hyperspectral imaging is 400-1000 nm, and the sample is 22 cm closer to the lens. For the spectral data of hyperspectral images, the successive projection algorithm (SPA) is used to complete the selection of characteristic wavelengths (617,675,818 nm). The prediction models of chlorophyll content based on PLS and SPA-PLS were established. The prediction sets r_p_ of the two models were 0.904 and 0.858, respectively, and RMSEP were 0.633% and 0.751%, respectively. Based on the characteristic wavelength ratio image, the recognition of peach decay area was completed. The results showed that the classification accuracy of healthy peach and diseased peach was 98.75% by using three characteristic wavelength ratio images. In the same year, Sun developed a hyperspectral imaging system with a 360° rotating platform to detect varying degrees of fungal infection in peaches (Sun et al., 2018). Such improvements are very helpful for the identification of decay occurring in different locations of peach fruit. In the study, the disease damage area was quantified into four categories, no decay, mild decay, moderate decay, and severe decay, with recognition rates of 95%, 66.29%, 100%, and 100%, respectively. The low recognition of mild decay can be attributed to the fact that the changes of internal physical and chemical properties of mild decay peaches are less affected by fungal infection, so they are not easy to be detected and identified.

Li et al. ‘s research on peach disease damage classification was done from the perspective of time (Li et al., 2021). The spectral reflectance of bruised fruits will decrease due to the presence of bruises, which is related to the firmness, density, titratable acid (TA), vitamin C and moisture after bruises. The reflectance of fruits in different time periods after bruises also changes, and as time increases, the color of the bruise position will gradually brown. Based on this, Li et al. used hyperspectral imaging technology to complete the classification of peach bruises at different time periods (12h, 24h, 36h, 48h). The wavelength range of hyperspectral imaging technology is 380-1080 nm, the spectral resolution is 2.8 nm, and the imaging environment is a closed darkroom environment. In the study, PCA was used to complete the screening of spectral characteristic wavelengths, and the average gray value feature of the bruise position was used as the image feature. A discriminant classification model based on PLS-DA and LS-SVM was established. The results showed that when the input variables were spectral features, the classification accuracy of PLS-DA model for peaches at 12,24,36 and 48 h after bruising was 96.67%, 96.67%, 93.33% and 83.33% respectively, and the correlation coefficient of training set was r_c_ = 0.928. Based on the LS-SVM algorithm, the correct classification of 12,24,36 and 48 h after bruising was 80%, 96.67%, 100% and 100%. This study can complete the detection of bruises as soon as possible. When the damage is softened and visible to the naked eye, the peach has lost its market value. Early detection of damage is necessary to reduce the loss. **Table 6** summarizes the research on fruit disease damage detection based on phenotypic information through hyperspectral images in recent years.

**Table 6:** Summary for the detection of fruit disease and damage based on phenotypic information using hyperspectral imaging.

Research object	Disease damage type	Wavelength range (nm)	Feature extraction	Method	Result	Ref.
Bi-colored peaches	Skin injury, scarring, insect, damage, puncture, injury, decay, disease, spots, dehiscent, scarring and anthracnose	325-1100 nm	PCA	Ratio images	Accuracy: 96.6%	(Li et al., 2016)
Peach	Early abrasions	325-1100 nm and 930-2548 nm	PCA	Image segmentation	Damage peach: 96.5% Non-destructive peach: 97.5%	(Li et al., 2018c)
Peach	Cold injury	400-1000 nm	/	MLP, ANN	Accuracy of cold-injured peaches: 95.8%	(Pan et al., 2016)
Honey peaches	Peach decay	400-1000 nm	SPA	PLS, SPA-PLS	PLS: r_p_=0.904, RMSEP=0.751% SPA-PLS: r_p_=0.858, RMSEP=0.633%	(Sun et al., 2017)
Peach	Fungal infection	400-1000 nm	SPA	PLS-DA	No decay: 95% Slight decay: 66.29% Moderate decay: 100% Severe decay: 100%	(Sun et al., 2018)
Peach	Bruises	380-1080 nm	PCA	PLS-DA, LS-SVM	12h, 24h, 36h, 48h after bruising; PLS-DA: 96.67%, 96.67%, 93.33% and 83.33% LS-SVM: 80%, 96.67%, 100%, 100%	(Li et al., 2021)

Through the summary of **Table 6**, we found that the types of disease damage detected based on hyperspectral images are abundant, including different types of defects located on the surface and inside. It is one of the advantages of hyperspectral imaging technology that the detection of multiple types of defects in the interior of the fruit is completed by the change of spectral characteristics presented by the acquired hyperspectral image, thus ensuring the integrity of the fruit. At the same time, we can also see that the detection objects in the reviewed related studies are concentrated on peaches. This may be because the volume of peaches is easier to complete the related research in the fruit of Prunoideae, and the peach fruit is more conducive to the progress of related research due to the low light reflection phenomenon on the surface.

### 3.3 Other related research based on fruit hyperspectral image

As a new detection technology, hyperspectral imaging technology is widely used by researchers because of its high efficiency, non-destructive and accurate detection characteristics. In addition to the above summary of the article, there are other applications related reports.

Munera ‘s team used hyperspectral images to identify nectarine varieties with similar appearance but different varieties (Munera et al., 2018). The imaging method of hyperspectral image is reflection imaging, and the wavelength range is 450-1040 nm. In the study, it was found that there were significant differences in the spectra of the two nectarines in the wavelength range near 680 nm and 970 nm, which indicated that there were differences in chlorophyll (Herrero-Langreo et al., 2011) and water content (Lu and Peng, 2006) between the two nectarines. The regression coefficient vector was used to determine the 14 optimal wavelengths, and a variety classification model based on PLS-DA was established. The average spectrum of a single fruit was used as the model input feature. The results show that the classification accuracy reaches 96.3%. The study also compared the variety classification based on hyperspectral images and color visual images. The results showed that the variety discrimination ability based on color vision image was low due to the high similarity of geometry and color of two nectarine varieties. In recent years, with the rapid development of image processing technology, spectral image and analysis technology, computer and data processing technology, hyperspectral imaging technology has been widely used in fruit phenotypic information and related research, such as maturity detection, fruit quality grading, disease damage identification and so on.

The image information and rich spectral data contained in hyperspectral images are the basis for the wide application of hyperspectral technology. For different use requirements and application fields, it is also necessary to select the wavelength range with the strongest correlation. Therefore, the choice of the optimal wavelength is also one of the problems faced by researchers. With the gradual improvement of machine learning theory, wavelength screening methods are also divided into two main methods: statistical methods and machine learning methods. There are more and more literatures using machine learning methods for data dimensionality reduction and model building, indicating that the combination of fruit phenotype information research based on hyperspectral image and intelligent computing is the general trend. However, there is no uniform and clear evaluation criteria for evaluating traditional modeling methods and machine learning-based modeling methods. In future research, the promotion of advanced technologies in practical production applications is also a field that cannot be ignored, pushing imaging equipment toward smaller, more sophisticated, lower cost, wider use of the environment, and faster, more accurate, and more efficient algorithms for wavelength selection and prediction models.

## 4 Phenotypic information acquisition and related applications based on multispectral image

In recent years, researchers have been exploring the use of spectral imaging equipment to obtain fruit phenotypic information, and based on the obtained fruit phenotypic information to achieve fruit disease damage (Kemps et al., 2010), fruit maturity and biochemical content (Li et al., 2018d) and other related applications. Multispectral images usually contain several to more than a dozen spectral bands, and some groups contained in organic substances, such as C-H, O-H, N-H, etc., these groups absorb energy in the spectral imaging bands used by multispectral imaging devices, resulting in changes in reflection or transmission spectra (Saranwong et al., 2004).

After the light beam emitted by the light source of the multispectral imaging device irradiates the surface of the fruit to be tested, part of the light beam will be reflected back after reaching the fruit to be tested, while the other part of the light beam will penetrate the fruit and scatter in different directions. Therefore, the imaging mode of the multispectral imaging device includes reflection imaging and transmission imaging. When the content of substance to be detected (SSC, TA, etc.) of fruit samples is different, different fruit samples will produce different spectral curves. Therefore, the corresponding relationship between the spectral data and the substance to be detected can be established according to the spectral characteristics of fruit samples, so as to realize the quantitative analysis of the content of the detected substance (Pissard et al., 2013). The researchers completed the measurement and analysis of relevant biomass, and combined the fruit biomass measurement research with practical application to explore the fruit maturity, disease damage, biochemical content and other related studies based on phenotype information.

### 4.1 Fruit maturity and biochemical parameters detection based on multispectral image

There are many criteria for determining the fruit maturity. These indicators include phenotypic information such as physical properties of the fruit (Herrero-Langreo et al., 2012) and biochemical parameters (Herrero-Langreo et al., 2011).Accurately grasping the fruit ripening stage is of great significance for guiding fruit picking time, post-harvest storage and fruit flavor. In recent years, significant progress has been made in the research on the acquisition of Prunoideae fruit phenotypic information based on multispectral images. The relevant research literature is summarized in **Table 7**.

**Table 7:** Summary table of fruit maturity and biochemical parameters detection based on multispectral image.

Research object	Wavelength coverage	Testing index	Processing method	Result	Ref.
Peaches	450, 689 nm;	Firmness	PCA+MLR	R^2 =^ 0.78	(Ruiz-Altisent et al., 2006)
Peach	632, 650, 670, 780, 850, 900 nm	SSC and firmness	Lorentzian distribution (LD), Gaussian distribution (GD), Exponential distribution (ED) MLR	r=0.949, SEP=1.56 N, r=0.970, SEP=0.69°Brix	(Muhua et al., 2007)
Peach	450, 675, 800 nm	Firmness	Ward classification algorithm, ANOVA, regressions	R/IR imaged>R image	(Lleó et al., 2009)
Cherry	550, 660, 735, 790 nm	Antioxidant Activity	XGBoost, RF, SVR, MLP	RMSE=6.74; MAPE=15.06	(Karydas et al., 2020)

Firmness is one of the most significant indicators of change during fruit ripening. Traditional methods for measuring fruit firmness such as micro-deformation measuring instruments and Magness-Taylor penetration/deformation resistance measuring instruments. However, these methods are harmful to fruit integrity and have the disadvantages of time-consuming and low accuracy. Spanish researcher M.Ruiz-Altisent first studied the relationship between peach fruit hardness and spectral wavelength to reflect the maturity of peaches (Ruiz-Altisent et al., 2006), and designed traditional measurement methods to verify the accuracy of optical measurement of peach fruit hardness. In the study, the spectral wavelengths of 450 nm and 680 nm were finally determined. The results of principal component analysis showed that the above two wavelengths were independent and complementary. The prediction model of spectral reflectance and hardness was established, the determination coefficient R^2^ = 0.78. At the same time, it was found that there were some differences in the setting of parameters when establishing prediction models for different varieties of peach fruits. The two spectral wavelengths obtained by principal component analysis are consistent with previous studies (Lu and Peng, 2006). In the study of Lu and Peng, it was found that the reflectance at 450 nm and 680 nm was related to carotenoid and chlorophyll content, respectively.

The researchers completed a test of maturity classification program based on multispectral images of peach fruits (Herrero-Langreo et al., 2011). The multispectral image data of peach fruits used in the verification were obtained from the same variety but different years. At the same time, the fruit segmentation problem of peach fruit multispectral image is optimized, and the triangle threshold segmentation algorithm is used to complete the segmentation of the region of interest of peach fruit infrared image. Compared with Otsu method, the triangle threshold segmentation method reduces the influence of highlight spots on fruits and irregularity in image background. Comparing the segmented images using the Otsu method and the triangle threshold segmentation method, in the experiment of peach fruit size estimation, the explained variance between the fruit measurement size and the image estimation size increased from 90% (Otsu) to 96%.

In the process of fruit ripening, the most intuitive and easy to observe phenomenon is that with the change of fruit maturity, fruit hardness will decrease significantly. In-depth exploration of the reasons we can know that this is due to the decomposition of certain biochemical substances within the fruit as the maturity changes. Therefore, in the detection of fruit maturity, the researchers in addition to the detection of hardness index, fruit chlorophyll content, fruit total soluble solids content (SSC), acidity, antioxidant components and other biochemical substances were also tried to measure. The traditional method for measuring the content of biochemical substances in fruit has the disadvantages of time consuming, damaging the integrity of fruit and large error. With the continuous exploration of researchers and technological progress, the detection technology based on various types of images for fruit internal biochemical content has gradually demonstrated the potential to replace traditional methods.

In addition to using multispectral images, some multispectral indices can also be used to detect fruit maturity. In subsequent reports, the researchers used four spectral indicators to detect peach fruit maturity (Lleó et al., 2011). It includes two new optical indices Ind1 and Ind2, and two previously used indices Ind3 (Sims and Gamon, 2002; Lleó et al., 2009) and IAD (Ziosi et al., 2008). The selection of the four optical indexes is within the range of chlorophyll absorption peak, and the change of optical index reflects the change of chlorophyll in fruit with maturity. The ability of four spectral indicators to distinguish fruit maturity was evaluated from two perspectives: (1) Maturity perception. The parameter Λ is introduced to evaluate the ability of each optical index to distinguish the maturity of peach fruit. The Λ parameter scores of the four indicators are as follows: Λ (Ind2) > Λ (Ind1) > Λ (IAD) > Λ (Ind3). (2) Robustness of indicators related to fruit convexity. The purpose of this comparison is to analyze which indicators are affected by fruit convexity. In the test of the effect of fruit convexity on spectral indexes, the results showed that only Ind1 was affected. In the study of L. Lleó, the intensity distribution of four optical indices in peach fruit images was obtained. It was found that the index Ind2 had the highest ability to distinguish maturity and was not affected by fruit convexity. Ind2 also allows the division of mature regions in the fruit and shows the evolution of these regions during ripening. In recent reports, researchers have tried to use changes in chlorophyll absorption index I_AD_ to reflect peach fruit maturity (Ziosi et al., 2008). I_AD_ as an optical index reflects the absorbance difference between 670 nm and 720 nm wavelengths. In the experiment, the linear regression method was used to establish the relationship between I_AD_ index and chlorophyll content, appearance color (L^*^, a^*^, b^*^), hardness, extractable juice and SSC/TA ratio of six different varieties of peach fruit. The results showed that the higher the I_AD_ value, the higher chlorophyll content, hardness, TA and b^*^ values, and the lower a^*^ value. I_AD_ was significantly positively correlated with chlorophyll (r^2^ > 0.8) and hardness (r^2^ > 0.6). The higher the I_AD_ value, the higher the TA content of the peach, while the SSC did not change, so the peach fruits with different I_AD_ values had different SSC/TA ratios. The results showed that I_AD_ index could divide peaches into different maturity groups according to chlorophyll content, SSC/TA and fruit firmness. They also found that such predictions were inaccurate for the internal quality of peach fruit, which was caused by the attenuation of light in peach fruit.

The imaging systems of the above researchers are mostly laboratory multispectral imaging systems. In the study of Karydas et al., multispectral imaging equipment was installed on an unmanned aerial vehicle platform to obtain four bands of cherry orchard aerial multispectral images (550,660,735,790 nm) (Karydas et al., 2020). The detection model of antioxidant components in cherry fruit was established by machine learning method, and the free radical scavenging activity (DPPH) of cherry fruit was analyzed. Three spectral indices were extracted based on multispectral images: normalized difference vegetation index (NDVI), carotenoid reflectance index 2 (CRI2) and anthocyanin reflectance index (ARI). Four machine learning algorithms are tested: extreme gradient boosting (XGBoost), random forest (RF), support vector regression (SVR) and multi-perceptron (MLP). The smaller RMSE and mean absolute percentage error (MAPE) obtained using the XGBoost algorithm in the study of the data obtained in 2018 were 6.74 and 15.06, respectively. In further studies, Karydas et al. were able to extend the prediction of DPPH for cherries throughout the orchard to accurately predict the maturity of cherries in different regions, guiding managers in the harvesting of cherries. The spectral indices (NDVI, CRI2, ARI) used in his research are the fusion of two or more bands of spectral data obtained by certain mathematical calculations (Crocombe, 2018). With the help of similar vegetation index (VI), the relevant research conclusions can be more obvious.

Multispectral imaging technology has been proved to be a feasible method for detecting fruit maturity and biochemical parameters. Compared with the traditional method, the detection method based on multispectral image has the advantages of high efficiency and non-destructive. However, the laboratory multispectral imaging equipment has higher requirements on the use environment, which also limits the application of multispectral image detection maturity and biochemical parameters in actual production. With the development of technology, such as UAV platform multispectral imaging equipment, portable multispectral equipment has been gradually developed, compared to the laboratory imaging equipment is small and easy to use features are more significant. Very useful for non-researchers (Li et al., 2018b). The research on fruit firmness, fruit SSC, size estimation and antioxidant content based on fruit multispectral images can provide guidance for fruit harvest time, fruit quality classification and fruit postharvest storage. It improves the quality of fruit, ensures its market value, and conforms to the development concept of precision agriculture.

### 4.2 Fruit disease damage detection based on multispectral image

Fruit disease damage is one of the most direct factors affecting fruit quality (Sun et al., 2019). The types of disease damage can be classified as existing in the fruit surface and fruit interior. The causes of diseases exist in many processes such as fruit growth, picking, and storage. Fruits with disease damage are more likely to rot, and if not treated in time, they can even cause lesions in other normal fruits. The multispectral image of fruit contains rich phenotypic information, and the detection of disease damage by multispectral image of fruit has been verified by many researchers.

Based on the phenomenon that the spectral characteristics of different disease damage tissues in fruit multispectral images are different, the detection of various types of fruit damage can be completed by using multispectral images. An improved enhanced GA-ANN is used to detect and classify different defect types in cherry multispectral images (Guyer and Yang, 2000). In this study, multispectral images of cherries were obtained by a non-portable multispectral imager and three spectral wavelengths of 680, 920 and 1120 nm were finally selected to complete the classification of seven different tissue types (dry crack, decay, mold, good tissue, background, stem or highlight). Researcher Daniel Guyer combines GA with ANN, and GA was used to optimize the weight of multi-layer feedforward artificial neural network. The results showed that the correct recognition rate of different tissue types reached 92%, while the correct recognition and accurate quantification of tissue types was only 72%. As can be seen from the results, more errors were made in the quantification process due to the similarity of the two types of defects, some moldy tissues were mistaken for rotten tissues. Sun et al. established a multispectral structured illumination reflection imaging system for the detection of early fungal infection in peach (Sun et al., 2019). The recognition rate of early fungal infection in peach fruit reached 98.6%, and the recognition rate of early infection without disease symptoms reached 97.6%. This study is the first time that the multispectral structured light reflection imaging system has been applied to detect fungal infection in peach fruits. Images of seven wavelengths between 690 and 810 nm at three different spatial frequencies of 60, 100 and 150 m^−1^ were obtained by the structured illumination reflection imaging system, followed by demodulation to obtain alternating component (AC) images and direct component (DC) images. Based on the acquired AC image, DC image and ratio image, three image classification methods, watershed algorithm, partial least squares discriminant analysis (PLS-DA) and CNN, were used to complete the detection and recognition of peach fruit lesions. The results show that AC images with wavelength and spatial frequency of 730 nm and 100 m^−1^ have high consistency, high detection rate and accuracy in disease region recognition and region estimation. In the horizontal comparison of classification algorithms, CNN is the best, followed by watershed segmentation algorithm.

**Figure 3:**
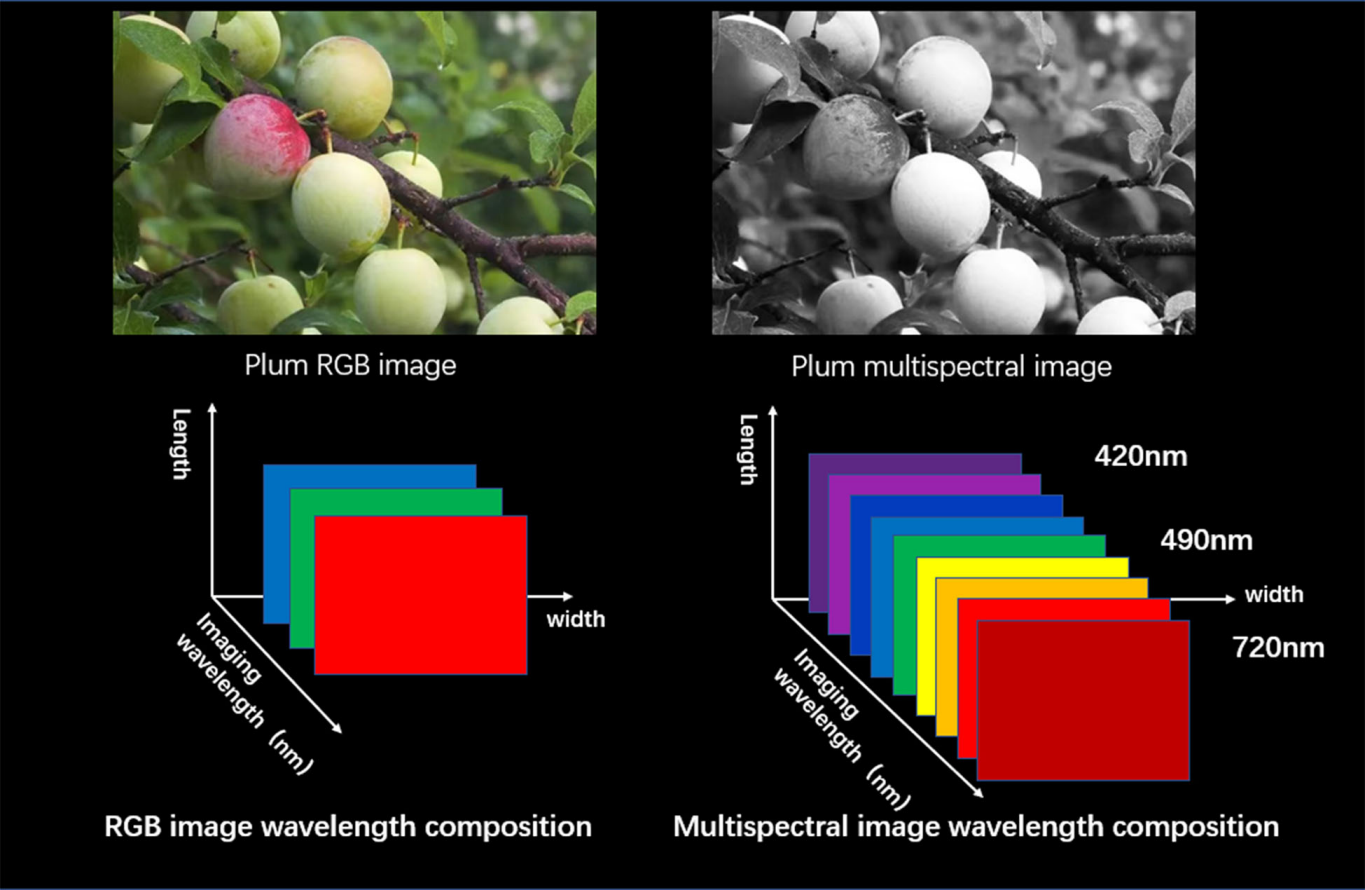
Wavelength composition of multispectral image.

Using multi-spectral imaging technology to detect fruit damage can efficiently and accurately complete the detection requirements, and give objective and quantitative evaluation (Alfatni et al., 2013). Therefore, fruit disease damage detection based on multi-spectral imaging technology has important research significance, and it is also very important to promote the automation, digitization and intelligent construction of fruit detection system. With the improvement of ANN algorithm, it has been widely used in image classification applications for disease damage types. The traditional image classifier needs to select feature vectors for the classification of fruit disease damage. If the feature selection is improper or insufficient, the classification accuracy will be directly affected. In comparison, the advantage of CNN model for image recognition is that its multi-level mechanism can extract and identify complex visual features, so the advantage of CNN is obvious. In addition, the defective tissues of fruits are often a combination of several types of defects, which complicates the identification and description of defects. Therefore, for each type of defect, the combination of the selected optimal wavelength and image processing operations can help to better complete the identification and detection. The wavelength composition of the fruit multispectral image is shown in **Figure 3**.

## 5 Phenotypic information acquisition and related applications based on other types of image

In the summary and review of the acquisition of phenotypic information of Prunoideae fruits based on multispectral images, hyperspectral images and RGB images and related applications, we also found that some other imaging techniques were used in the related research of phenotypic information acquisition of Prunoideae fruits, including thermal imaging technology, computer tomography (CT) technology, laser-light backscattering imaging (LLBI) and so on. These imaging techniques have not been widely used in the related research of the Prunoideae fruit, but they cannot be ignored. In the following sections, we analyze and review the cited literature according to different imaging techniques.

### 5.1 Phenotypic information acquisition based on thermal imaging technology and related research

The thermal imaging equipment uses the infrared detector and the optical imaging mirror to receive the infrared radiation energy of the measured target, and reflects the energy distribution to the photosensitive element of the infrared detector, so as to obtain the thermal image of the measured target. The obtained thermal image corresponds to the thermal distribution of the measured object (Lee et al., 2019). The imaging methods of thermal imaging equipment include active system and passive system. Compared with passive system, active system usually includes heating or cooling system.

Cherry is a kind of fruit which is sensitive to the surface temperature and humidity. Fruit cracking caused by abnormal changes in fruit surface temperature and humidity will seriously affect its market value. Osroosh et al. used thermal imager-RGB image system to detect the surface temperature of cherry fruit and used microclimate information detection system to verify the results (Osroosh and Peters, 2019). The thermal imaging equipment was installed at a distance of 20 cm from the target cherry. In the experiment, a simulated rainfall system was also built to change the surface humidity of the cherry fruit. They also developed a custom computer vision algorithm to recognize cherry fruits in thermal images and RGB images, and completed the extraction of fruit surface temperature. In the study, they found that the surface temperature of cherry fruits was highly correlated with the surface temperature of leaves (R^2^ > 0.89). The final experimental results show that it is feasible to detect the surface temperature of cherry fruit using a system based on low-resolution thermal RGB images. They also established the normalized temperature index (NTDI and NRTI) to quantify fruit surface humidity levels.

In a recently reported study, researchers developed two models for predicting cherry surface humidity based on thermal-RGB images and weather sensing systems (Ranjan et al., 2022). The input data of the first model is weather sensor data, and the input data of the other model combines the fruit surface temperature obtained from thermal image data. An automatic custom image processing algorithm was developed for fruit recognition and surface temperature extraction, and the radiation calibration equation was used to correct the temperature data. In the experiment, two varieties of cherry fruits were used to complete the prediction of fruit surface humidity. The results showed that the correlation between the measured and predicted values of humidity was R^2^ = 0.80 and R^2^ = 0.86, respectively. Compared with other imaging technologies, thermal imaging technology can accurately provide the temperature information of the measured target, which is an advantage that other types of images do not have. Especially in the exploration of temperature and fruit phenotype research applications, has an irreplaceable role. However, the use of thermal imaging equipment in outdoor environments is strongly affected by light, which will have a certain impact on its accuracy.

### 5.2 Phenotypic information acquisition based on computed tomography imaging technology and related research

Computer tomography (CT) has become one of the mature non-destructive technologies for measuring the external morphological characteristics and internal defect detection of agricultural products (Arendse et al., 2018). The CT technology ray includes X-ray, γ-ray, ultrasound, etc. CT technology can reflect the density change of the measured sample. The density and absorption coefficient of the measured sample will lead to the attenuation of the ray during the penetration process. The CT image can reflect the density change of the measured object through the gray value of the pixel. The image is white, indicating high density, and black indicates low density.

Kritzinger et al. used X-ray CT technology to detect the occurrence of fruit core cracking during plum fruit development (Kritzinger et al., 2017). They selected six plum varieties to explore the causes of fruit core cracking, and randomly selected measurement targets in the orchard for X-ray CT scanning. The acquisition process of CT images developed from the inner epidermis until the fruit core was completely hardened. The results showed that due to the influence of temperature changes, the growth of the inner epidermis of the fruit was affected, and the incompletely hardened inner epidermis was affected by the tension of the pulp, resulting in cracking. In their research, CT technology accurately reflected the process of fruit core hardening at different stages, thus accurately discovering the occurrence of fruit core cracking.

In a study to determine whether 1-Methylcyclopropene treatment of apricot fruit is beneficial for fruit preservation, X-ray CT technology was used to detect the occurrence of voids inside the fruit (Karmoker et al., 2018). The obtained fruit CT images are divided into high-density regions (−200 ~ +350HU) and low-density regions (−900 ~ −200HU) to detect voids. The changes of ethylene content in fruits treated with conventional methods and 1-Methylcyclopropene during storage were compared. The results showed that the apricot fruit pretreated with 1-Methylcyclopropene effectively inhibited the production of ethylene. From the CT image of the fruit, it can be seen that the inside voids the treated apricot fruit is less than that of the apricot fruit preserved by the conventional method. These results show that CT imaging technology has significant advantages in the detection of fruit internal.

### 5.3 Phenotypic information acquisition based on laser-light backscattering imaging technology and related research

Laser-light backscattering imaging (LLBI), as a low-cost imaging technology, realizes the detection and analysis of targets by using the principles of light absorption, scattering and image processing in the visible and near-infrared electromagnetic spectrum. When a beam of bright light is irradiated to the fruit surface, most of the light will transmit to the fruit tissue, and the other part will diffuse to the fruit surface (Adebayo et al., 2016). Through the interaction between light and the object to be measured, useful information about the structure and composition of the object to be measured is provided for quality analysis of fruits.

In 2016, a quality evaluation study using LLBI technology to detect plum fruit during tree development and storage was reported (Rezaei Kalaj et al., 2016). In this study, two different wavelengths of light (532,785nm) were used to detect two different varieties of plum. The full width at half maximum (FWHM) value was obtained by radial backscattering profile calculation, and this value was tried to establish with fruit hardness, SSC, dry matter content and normalized anthocyanin index. The results showed that the decrease of FWHM532 was closely related to the increase of anthocyanin content during fruit development. In addition, the increase of FWHM785 was closely related to the decrease of flesh firmness during fruit development and storage. Their results show that it is feasible to use the appropriate wavelength LLBI technique to nondestructively detect the oxidation resistance and firmness of plum fruit.

In a recent study, LLBI technique was used to predict the quality characteristics and maturity of apricot fruit (Mozaffari et al., 2022). Different from previous studies, the wavelength of 650 nm was used to obtain backscatter images of six ripening stages of apricot fruit. They used Otsu and first inflection point techniques to segment the image and extract spatial domain features from it. They established three prediction models (ANN, PLSR, PCA-ANN) to predict the hardness and SSC of apricot. The results show that the R^2^ based on ANN prediction model is the highest and RMSE is the lowest. The results of hardness and SSC were R^2^_CV_ = 0.974, RMSE_CV_ = 3.482 and R^2^_CV_ = 0.963, RMSE_CV_ = 1.146, respectively.

## 6 Discussion

At present, the acquisition of fruit phenotypic information based on image technology and its related research have made remarkable progress under the efforts of researchers. In the review of past research, our research based on image type is divided into three types. Here, we discuss the related research of three image types.

(1) Fruit phenotypic information acquisition and related research based on RGB images: Firstly, from the perspective of images, RGB images only contain image information compared with the former two, and do not involve spectral data. In terms of phenotypic information acquisition, only fruit color features, texture features and geometric morphological parameters based on image information can be extracted. However, the convenience of RGB image acquisition is one of its most significant advantages.

The convenience of RGB image acquisition is one of its most significant advantages. However, with the popularity of various imaging devices, the obtained RGB images have differences in pixel resolution, image size, etc. These differences should be solved in subsequent image processing. In addition, the acquisition of RGB images should also take into account the automatic preprocessing of the image during the acquisition of the image by the smartphone, such as the adjustment of exposure time and contrast, which will lead to the acquisition of RGB images can not accurately reflect the color characteristics of the target object.

(2) Fruit phenotypic information acquisition and related research based on hyperspectral images: Compared with multispectral images, hyperspectral images have become one of the research hotspots with richer spectral data. The spectral resolution of hyperspectral imaging equipment is nanoscale, and the hyperspectral image obtained often contains hundreds of thousands of wavelengths. Therefore, the rich spectral data makes the hyperspectral image more comprehensive and accurate in the process of obtaining fruit phenotypic information, which makes it widely studied in the fields of fruit maturity detection, fruit quality grading, disease damage identification quantification and so on.

Removing redundant hyperspectral data is also one of the hot issues for researchers in related research using hyperspectral images. With the improvement of artificial intelligence theory, there is also great potential in feature wavelength selection algorithms, such as UVE, SVE, etc., which have achieved good results. It should be mentioned that in most of the studies reviewed, hyperspectral imaging equipment is used in a laboratory environment, and some researchers have also used portable spectral imaging equipment for research. However, further improvement is needed to balance image quality and cost issues.

(3) Fruit phenotypic information acquisition and related research based on multispectral images: Fruit multispectral images are used by researchers in fruit maturity, biochemical parameter detection, disease damage detection and other fields by virtue of the image information and spectral data contained in the image. On the one hand, the band range of multispectral images often contains only a few to a dozen, and there is a defect that the spectral data acquisition is not comprehensive, which limits the researchers to obtain various phenotypic information of fruits through spectral data and complete related applications. On the other hand, the low number of spectral data in multispectral images also reduces the complexity of data dimensionality reduction and further processing in the later stage. From the perspective of multi-spectral imaging equipment, the low cost and portability of multi-spectral imaging equipment are its most significant features. Considering that the imaging environment is mostly outdoor, sunlight will have a certain impact on multispectral images, so multispectral imaging equipment should be further improved in terms of resolution and anti-interference ability.

(4) In the review and summary of other imaging technology, we mentioned thermal imaging technology, CT imaging technology, LLBI technology. The images obtained by these imaging techniques can provide data in different dimensions for the acquisition of fruit phenotypic information and subsequent research applications. The acquisition of fruit surface temperature information by thermal imaging technology is irreplaceable in the study of avoiding fruit cracking due to abnormal surface temperature. CT imaging technology and LLBI technology can accurately reflect the internal quality of fruits by means of X-ray and laser beam penetration measurement in the detection of internal phenotypic information of fruits.

Finally, with the development of image processing technology and related target recognition and segmentation algorithm, the acquisition of fruit phenotype information based on RGB image and related research is still one of the important areas. Researchers have used a variety of neural network algorithms to complete research on maturity, fruit quality, disease damage and other related research based on RGB images. In this review article, we do not deeply explore the related algorithms used by researchers, but it can be concluded that with the continuous improvement of related algorithms, deep learning shows significant advantages over traditional algorithms.

## 7 Conclusions

We tried to review the acquisition of fruit phenotypic information and related research based on image technology. Image types mainly include multispectral, hyperspectral and RGB images. Then, according to the research purpose, the research based on each type of image is introduced, including the imaging equipment parameters, image analysis technology and research results, etc. When the actual design is based on different types of images to complete the application of fruit detection, due to the differences, it may be necessary to readjust the various parameters of the image analysis algorithm and combine various technologies. In addition, the specificity of the imaging equipment and imaging environment used in the references leads to its popularization to be improved. In some researches based on natural light and outdoor environments, the preprocessing algorithms proposed for other interference light sources are worthy of reference, which can significantly reduce the impact of noise in target fruit recognition and detection, thereby improving the accuracy of research. In order to further promote the application of image-based fruit detection technology in actual production, it is necessary to further improve the portability of imaging equipment and coordinate the cost problem. Moreover, it can be seen from the environmental specificity of previous studies that the existing analysis algorithms have poor universality. Therefore, the development of new algorithms to achieve high efficiency, high accuracy and strong adaptability is of great significance for the promotion of this technology in practical applications.

## Data availability statement

The original contributions presented in the study are included in the article/Supplementary material. Further inquiries can be directed to the corresponding author.

## Author contributions

XL collected and analyzed references. XL and NL prepared the review and wrote the manuscript; YH and XJL offered much help in the process of revision. ZR provided financial supports. All authors read and approved the final manuscript.
